# Neuron Regeneration and Proliferation Effects of Danshen and Tanshinone IIA

**DOI:** 10.1155/2011/378907

**Published:** 2010-12-01

**Authors:** Jui-Lung Shen, Yueh-Sheng Chen, Jing-Ying Lin, Yun-Chen Tien, Wen-Huang Peng, Chia-Hua Kuo, Bor-Show Tzang, Hwai-Lee Wang, Fuu-Jen Tsai, Ming-Chih Chou, Chih-Yang Huang, Chien-Chung Lin

**Affiliations:** ^1^Department of Dermatology, Taichung Veterans General Hospital, Taichung 40705, Taiwan; ^2^Institute of Medicine, Chung Shan Medical University, Taichung 40705, Taiwan; ^3^Graduate Institute of Chinese Medical Science, China Medical University, Taichung 404, Taiwan; ^4^Department of Medical Imaging and Radiological Science, Central Taiwan University of Science and Technology, Taichung 40705, Taiwan; ^5^Graduate Institute of Chinese Pharmaceutical Sciences, China Medical University, Taichung 40705, Taiwan; ^6^Laboratory of Exercise Biochemistry, Taipei Physical Education College, Taipei 105, Taiwan; ^7^Institute of Biochemistry and Biotechnology, Chung Shan Medical University, Taichung 40705, Taiwan; ^8^Department of Pediatrics, Medical Research and Medical Genetics, China Medical University, Taichung 40705, Taiwan; ^9^School of Medicine, Chung Shan Medical University, Taichung 402, Taiwan; ^10^Department of Surgery, Chung Shan Medical University Hospital, Taichung 402, Taiwan; ^11^Graduate Institute of Basic Medical Science, China Medical University, 40705 Taichung, Taiwan; ^12^Department of Health and Nutrition Biotechnology, Asia University, Taichung 413, Taiwan; ^13^Orthopaedic Department, Taichung, Armed Forces General Hospital, Taichung 41152, Taiwan; ^14^Department of Materials and Science and Engineering, National Chung Hsing University, Taichung 402, Taiwan

## Abstract

This study evaluates the proliferative effects of danshen and its monomer extract, tanshinone IIA, on Schwann cell proliferation. A piece of silicone rubber was guided across a 15-mm gap in the sciatic nerve of a rat. This nerve gap was then filled with different concentrations of danshen (0–100 mg/mL). The results showed that danshen increased the expressions of uPA, cyclin D1, E and ERK, JNK, and P38 MAP kinases via the FGF-2 signaling pathway in a dose-dependent manner. RSC96, Schwann cells were also administered with danshen (0, 20, 40, 60, 80, and 100 *μ*g/mL) and tanshinone IIA (0, 2, 4, 6, 8, and 10 *μ*g/mL). In lower concentrations, danshen and tanshinone IIA exhibited an apparent effect on Schwann cells. Similar effects were also demonstrated in the FGF-2-uPA regulating cascade and cell cycle proliferative protein results. Schwann cell migration was elevated as well. We used MAPK-signaling chemical inhibitors and identified the proliferative effects of danshen and tanshinone IIA as MAPK-signaling dependent. The results from the *in vitro* systems indicate that danshen and tanshinone IIA can be used to induce Schwann cell proliferation, and *in vivo* results potentially suggest that danshen and tanshinone IIA might enhance neuron regeneration.

## 1. Introduction

The central and peripheral nervous systems are composed of neurons with different anatomical structures and regeneration ability. Neuron injury elicits a cascade of physiological responses that stimulate nerve cell regeneration. In mammals, the central neurons do not have a myelin sheath and therefore do not regenerate. Neurons in the peripheral nervous system are surrounded by a myelin sheath and can undergo regrowth [1]. The ability of neurons to regenerate is due to intrinsic neuronal activities and the presence of surrounding nonneuronal components, such as Schwann cells. Schwann cells play a crucial role in endogenous repair of peripheral nerves because of their ability to dedifferentiate, migrate, proliferate, express growth promoting factors, and myelinate regenerating axons. These cells migrate from the periphery into the injury site, where they apparently participate in endogenous repair processes [2]. If regenerating axons gain a distal nerve, they elongate within the endoneurial tubes, in association with the Schwann cells and the basal lamina, constituting regenerative units [3]. Schwann cells also produce some growth factors which are crucial for peripheral nerve repair. Fibroblast growth factor-2 (FGF-2) has a neruotrophic effect on peripheral nerve regeneration by maintaining cell survival [4] and stimulating cell proliferation [5]. Interestingly, up-regulation of FGF-2 in Schwann cells occurs only when the peripheral nerves are injured [6]. Furthermore, urokinase plasminogen activator (uPA) is involved in tissue regeneration. uPA cleaves plasminogen to plasmin to promote extracellular matrix (ECM) degradation for cell proliferation during the recovery from injury [7]. Therefore, the ability of Schwann cells to promote regeneration in peripheral neurons has led to an increasing interest in using Schwann cells to study peripheral nervous system repair. Enhancing the proliferative effect of Schwann cells might be another potential approach to induce neuron regeneration.

 Biomaterials combined with Chinese herbal medicine have been applied in the study of nerve regeneration. For example, a silicon rubber chamber filled with Schwann cells bridged a 15-mm defect in rat sciatic nerves. Several Chinese medicines have been identified to enhance neuron regeneration. Therefore, targeting Schwann cells with herbal medicines to induce neuron regrowth may be a possible therapeutic approach for treating injured nerves. danshen (*Salvia miltiorrhiza*) is a Chinese herb commonly used in mainland China for the treatment of atherosclerosis-related disorders such as cardiovascular and cerebrovascular diseases. In traditional Chinese medicine, danshen is believed to remove blood stasis and promote blood flow, stimulate menstrual bleeding, relieve pain and inflammation, and reduce stress [8]. Danshen has been shown to have anticancer effects [9] and inhibit oxysterol-induced endothelial cell apoptosis [10]. Whether danshen has a nerve regenerating effect and growth enhancing effect on Schwann cells is largely unknown. However, tanshinone IIA, a monomer extracted from danshen, has been reported to have neuroprotective effects on neonatal hypoxia-ischemia brain damage [11] and in transient focal cerebral ischemia [12]. Therefore, we used an *in vivo* and *in vitro* system to compare the effects of danshen and tanshinone IIA on Schwann cell proliferation neuron regeneration. In the animal model, danshen at different concentrations (0, 20, 40, 60, 80, and 100 mg/mL) was injected into dissected rat sciatic nerves. In the *in vitro* model, Schwann cells were treated with different concentrations of danshen (0, 20, 40, 60, 80, and 100 *μ*g/mL) and tanshinone IIA (0, 2, 4, 6, 8, and 10 *μ*g/mL).

## 2. Materials and Methods

### 2.1. Danshen Extraction

Danshen (Ko Da pharmaceutical company, Taoyuan, Taipei County, Taiwan) powder was weighed and extracted using sterilized distillated deionized water. The concentrations used in the animal model were 0, 20, 40, 60, 80, and 100 mg/mL. The doses for the *in vitro* model were 0, 20, 40, 60, 80, and 100 *μ*g/mL for Schwann cell treatment. All of the solutions were stored at −20°C. Pure danshinone IIA (Jing-Ming chemical industry company, Taichung, Taiwan) was dissolved in dimethyl sulfoxide (DMSO). The following concentrations were prepared: 2, 4, 6, 8, and 10 *μ*g/mL.

### 2.2. Animal Model and Treatments

The surgery was performed as described in a previous study [13]. Twenty-four adult Sprague-Dawley rats (National Science Council) weighing 220 ± 20 gm, underwent silicone chamber placement. Animals were equally divided into six groups. For each animal, the right legs received experimental treatment and the left legs were used as the control. In the first group, the chambers were filled with saline only. 

The chambers in groups 2~6 were filled with different concentrations of danshen at 20, 40, 60, 80, and 100 mg/mL, respectively. 

The chamber lumen volume was 25.5 *μ*L. Danshen was injected through a precooled micropipette into the lumen by passing the tip of the needle into the silicone rubber chamber. Loading was performed as slowly as possible to prevent the formation of air bubbles. The distal stump was then secured at the other end of the chamber. Both the proximal and distal stumps were secured to a depth of 1 mm into the chamber, leaving a 15 mm gap between the stumps. The muscle layer was reapproximated using 4–0 chromic gut sutures, and the skin was closed with 2–0 silk sutures. All animals were housed at 22°C and 45% humidity with a 12-hour light/dark cycle. They had free access to rodent chow and water at libitum. After eight weeks, animals were anesthetized by isoflurane (Abbott Laboratories Ltd., Queenborough, Kent, England), the nerves were reexposed and the chambers across the 15 mm gap were examined for nerve regeneration. All animals were maintained in facilities approved by the China Medical University for Accredited Laboratory Animal Care, according to the regulations and standards of the National Science Council of Health of the Republic of China.

### 2.3. Cell Culture and Treatments

RSC96 cells were purchased from American Type Culture Collection (ATCC) and were cultured in Dulbecco's modified Eagle's medium (DMEM) supplemented with 10% fetal bovine serum (FBS), 4 mM L-glutamate, 1.5 g/L sodium bicarbonate, and 1% Nonessential amino acids (NEAA) in humidified atmosphere of 5% CO_2_ and 95% air. After 4-hour serum starvation, the cells were treated with danshen and tanshinone IIA at concentrations of 0, 20, 40, 60, 80, and 100 *μ*g/mL and 2, 4, 6, 8 and 10 *μ*g/mL, respectively. The incubation was continued for another 16 h, according to our preliminary test and then the cells were harvested and extracted for analysis.

### 2.4. Inhibitors

RSC96 Schwann cells were treated with several inhibitors, including SB203580 (p38 MAP kinase inhibitor; Promega, Madison, WI, USA), U0126 (MEK1 and MEK2 inhibitor; Promega, Madison, WI, USA), and SP600125 (JNK inhibitor; Promega, Madison, WI, USA). The final concentration of each inhibitor was 0.5 *μ*M, according to our previous experiments.

### 2.5. Western Blotting

Sciatic nerve sections were extracted from the middle of the regenerated nerve in the chamber. Cultured RSC96 cells were scraped and washed. The procedure was described in our previous study [5]. Proteins were then separated by 12% gradient SDS-PAGE and transferred to nitrocellulose membranes. Nonspecific protein binding was stopped in blocking buffer (5% milk, 20 mM Tris-HCl, pH 7.6, 150 mM NaCl, and 0.1% Tween 20) and blotted with specific antibodies in the blocking buffer at 4°C overnight. For repeated blotting, nitrocellulose membranes were stripped with Restore Western blot stripping buffer (Pierce Biotechnology, Inc, Rockford, IL, USA) at room temperature for 30 min.

### 2.6. MTT Assay

Cell viability was estimated using a colorimetric assay based on tetrazolium dye (MTT) conversion into a blue formazan product. The procedure was described in our previous study (Chen et al., 2007). After harvesting and washing twice with PBS, the cells were cultured in phenol red-free DMEM (1 mL) with MTT (0.5 mg/mL) at 37°C for 4 h. The cells were incubated in iso-propenol (1 mL) with shaking for 10 min. They were then aspirated and measured spectrophotometerically at 570 nm.

### 2.7. Wound Healing

Cells were initially seeded uniformly in 60-mm culture plates with an artificial “wound” carefully created at 0 h, using a sterile P-200 pipette tip to scratch on the subconfluent cell monolayer to make a 1.0 mm gap. After 24-hour exposure to different concentrations of danshen and tanshinone IIA, cells were calculated by counting the number of cells that had advanced into the cell-free space randomly selected from the initial wound border area. Photographs were taken of the wounded regions using an inverted Olympus microscope.

### 2.8. Zymograthphy

MMP-2 and MMP-9 activity was determined using gelatin zymography. RSC96 cells were treated with different concentrations of danshen and tanshinone IIA which were already showed. Cell medium was collected after incubation for 16 hours. Sample medium was electrophoresed on an 8% polyacrylamide gel containing 0.1% gelatin. After electrophoresis, the gel was washed for 30 min 2 times in washing buffer (2.5% Triton X-100). The gel was then incubated in incubation buffer (1% NaN_3_; 2 M Tris-HCl, pH 8.0; 1 M CaCl_2_) at 37°C for 48 hours with shaking and subsequently stained with Coomassie blue. The presence of MMP-2 and MMP-9 gelatinolytic activity was identified as clear bands on a blue background after destaining.

### 2.9. Migration Assay

 We used a Boyden chamber and polyvinyl-pyrrolidone-free polycarbonate membranes with 8 *μ*M pores (Neuro Probes, Inc.) for the migration assay. The bottom wells of the chamber were filled with 10% FBS DMEM medium. The wells were covered with a membrane sheet. The membranes were stained with Giemsa stain (Sigma). Cells that migrated through the membrane were counted using a counting grid fitted into the eyepiece of a phase contrast microscope.

### 2.10. Statistical Analysis

Statistical differences were assessed using one way-ANOVA. *P* < .05 was considered statistically significant. Data are expressed as the mean ± SEM.

## 3. Results

### 3.1. Proliferative Effects of Danshen on Damaged Peripheral Nerves in Animals

**Figure 1:**
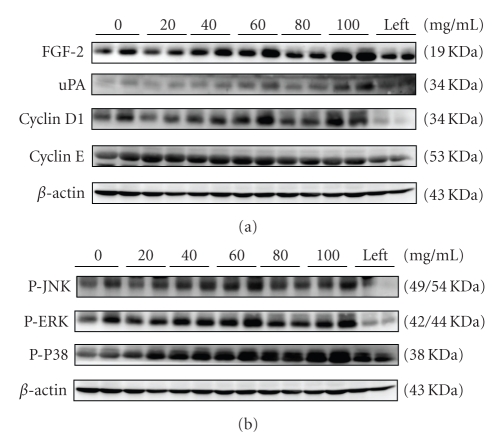
The regeneration of dissected sciatic nerves in the chambers filled with danshen and tanshinone IIA. The sciatic nerves from the chamber in rats with surgery were taken and the FGF and cell cycle signaling activities (a) and MAPK signaling activities (b) were examined using western blotting analysis. The chambers in the right legs were filled with various concentrations of danshen and tanshinone IIA as indicated. The left represents the sciatic nerves treated with saline from the left legs of each animal as an experimental control. *β*-actin was used as a loading control.

To investigate whether danshen can promote damaged nerve regeneration, various concentrations of danshen were injected into silicon chambers connecting the distal and proximal stumps of sciatic nerves. Danshen activated the FGF-signaling pathway as evidenced by increased levels of FGF-2 and uPA. The cyclin proteins, D1, E, were also increased (Figure 1(a)). To identify the role of MAPK signaling in danshen-induced nerve cell proliferation, we examined the MAPK signaling activities in regenerated nerves and found that the levels of phosphorylated ERK, JNK, and P38 were increased (Figure 1(b)). These observations indicate that danshen might promote nerve regenerative markers, such as FGF-signaling, cell cycle activity, and MAPKs, ERK-, JNK- and P38-signaling pathways.

### 3.2. Dose-Dependent Proliferation of RSC Cells by Danshen and Tanshinone IIA In Vitro

**Figure 2:**
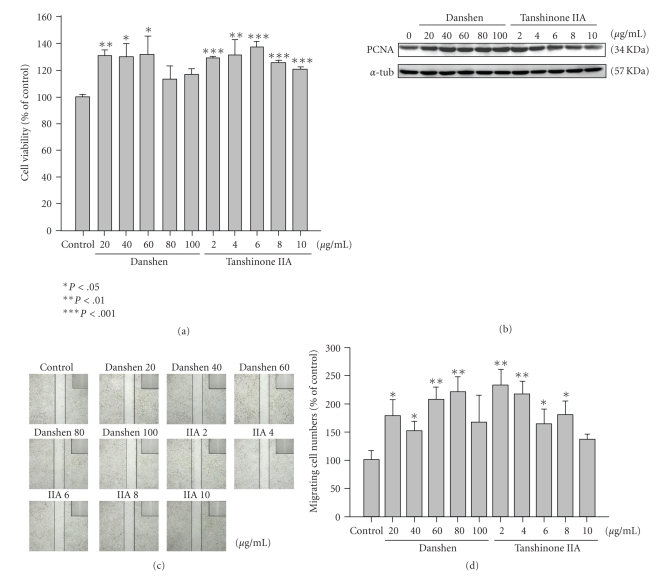
Proliferative effects of danshen and tanshinone IIA on RSC96 cell viability and migration. RSC cells were incubated with different doses of danshen and tanshinone IIA as indicated. Cell viability was measured using MTT assay (a), and western blotting analysis (b); cell migration was measured using wound healing analysis (c). The procedures are described in the materials and methods section. The wound healing data were quantified by measuring the number of cells that had advanced into the cell-free space (d). Data are shown as the mean of three independent experiments ± SE. *, **, and *** denote significant differences between experimental and control values, with *P* < .05, *P* < .01, and *P* < .001, respectively. *α*-Tubulin was used as a loading control. PCNA: proliferating cell nuclear antigen.

At 16 h of culture, MTT assay revealed that danshen (20~100 *μ*g/mL) and tanshinone IIA (2~10 *μ*g/mL) had a significant proliferative effect on RSC cells in a dose-dependent manner using MTT assay for 24 h culture. However, cellular viability was no effect with high concentrations of danshen (Figure 2(a)). The level of PCNA (proliferation cell nuclear antigen) protein expression in RSC cells also increased after treatment with danshen and tanshinone IIA (Figure 2(b)). Therefore, we performed an *in vitro* wound healing assay to evaluate the migration potential of Schwann cells treated with various concentrations of danshen and tanshinone IIA and found that the cell migration was promoted by danshen and tanshinone IIA treatment (Figures 2(c) and 2(d)). These observations provide that danshen and tanshinone IIA not only induce Schwann cell proliferation but also migration, thereby enhancing nerve regeneration. 

### 3.3. Proliferative and Migrative Effects of Danshen and Tanshinone IIA on Schwann Cells

**Figure 3:**
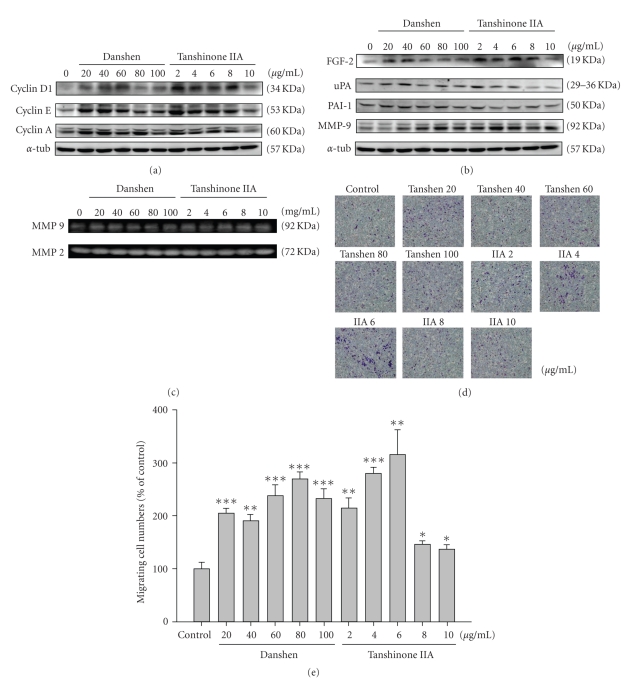
Exaimination of cell cycle signaling (a), activated FGF signaling (b, c), and Schwann cell migration of danshen and tanshinone IIA on RSC. RSC cells were incubated with different doses of danshen and tanshinone IIA as indicated for 16 hrs. Cells were then lysed. Protein was extracted and analyzed using western blotting. *α*-tubulin was used as a loading control. (c) Matrix metalloprotease-9 and -2 enzyme activity was examined using zymography. (d) The Schwann cell migration was examined using the Boyden chamber system. The number of cells that had migrated were quantitated using the mean value of 10 microscopic fields in every condition. *, **, and *** denote significant differences between experimental and control values, with *P* < .05, *P* < .01, and *P* < .001.

We evaluated the proliferative effect of danshen and tanshinone IIA on the regenerative ability of Schwann cells, which play an important role in nerve regeneration. As shown in Figure 3(a), representing the protein level results, administration of danshen and tanshinone IIA increased the level of FGF-2, MMP 9, and uPA but decreased the level of PAI-1. The two compounds also promoted cell cycle activity by increasing the levels of cyclin proteins, D1, E, and A (Figure 3(b)). Zymography revealed MMP 9 enzyme activity increased after danshen and tanshinone IIA treatment (Figure 3(c)). Using the Boyden chamber to observe Schwann cell migration, we found that cell migration increased after danshen and tanshinone IIA treatment (Figure 3(d)). These results provide evidence that danshen and tanshinone IIA have proliferative and migrative effects on Schwann cells. The maximum danshen and tanshinone IIA effects were observed at concentrations of 20–60 *μ*g/mL and 2–8 *μ*g/mL, respectively. Some of the effects reversed back to the basal level at doses of 80, and 100 *μ*g/mL in Danshen 10 *μ*g/mL in Tanshinone IIA, indicating that danshen and tanshinone IIA are toxic at those concentrations.

### 3.4. RSC Cell Proliferation Enhanced by Danshen and Tanshinone IIA Is MAPK Signaling-Dependent In Vitro

**Figure 4:**
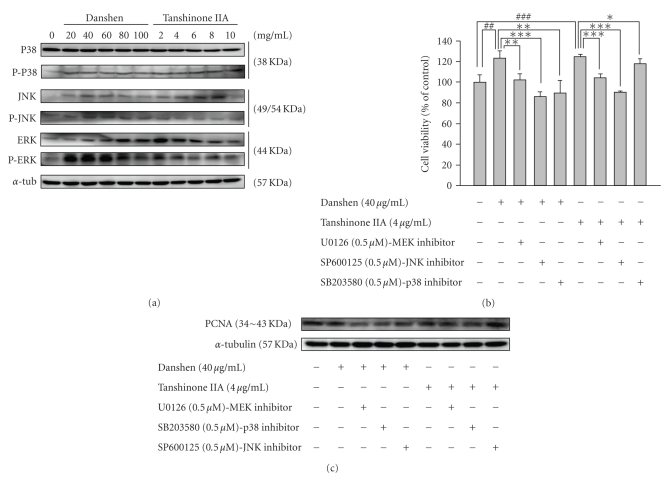
The proliferative effect of danshen and tanshinone IIA on RSC96 cell viability has ERK-, JNK- and P38-signaling dependent. The RSC96 cells were treated with various doses of danshen and tanshinone IIA. The treatments and procedures are the same as those described for Figure 3. The MAPK-signaling activities were measured using western blotting (a). The RSC96 cells were treated with danshen and tanshinone IIA at concentrations of 40 *μ*g/mL and 4 *μ*g/mL, respectively, and MAPK signaling inhibitors as indicated. Cell viability was examined using MTT assay (b); the cell proliferation was analyzed using western blot analysis of PCNA (c). *α*-tubulin was used as a loading control. U0126, SB203508, and SP600125 are inhibitors of MEK, P38, and JNK, respectively. ^##^ and ^###^ denote significant differences between experimental and control values with *P* < .01, and *P* < .001. *, **, and *** denote significant differences between danshen and tanshinone IIA values with *P* < .05, *P* < .01, and *P* < .001.

Intracellular MAPK signaling components, ERK1/2, JNK1/2, P38, and their phosphorylations were measured by western blotting. Our data showed, danshen and tanshinone IIA treatment increased the level of ERK1/2, JNK1/2 and P38 phosphorylation. Treatment also increased the total protein levels of ERK1/2, JNK1/2, but did not increase that of P38. However, danshen and tanshinone IIA at concentrations of 100 *μ*g/mL and 10 *μ*g/mL, respectively, reduced the level of phosphorylation and total protein levels of ERK1/2, JNK1/2, and P38 (Figure 4(a)). To further investigate the roles that MAPKs play in danshen and tanshinone IIA-induced cell proliferation, RSC cells were pretreated with pharmacological inhibitors of individual MAPKs, followed by incubation with danshen and tanshinone IIA at concentrations of 40 *μ*g/mL and 4 *μ*g/mL, respectively, for 24 h. All inhibitors, U0126 (a potent inhibitor of MEK), SP600125 (a potent inhibitor of JNK), and SB203580 (a potent inhibitor of P38). MTT assay revealed that all three MAPK inhibitors significantly decreased the level of danshen and tanshinone IIA-induced cell viability (Figure 4(b)). PCNA protein levels in danshen and tanshinone IIA-treated RSC cells that had been pretreatment with individual inhibitors were also examined. We found that the PCNA levels were significantly reduced after pretreatment with inhibitors of ERK1/2, JNK1/2, and P38 (Figure 4(c)). These results indicate that signaling of MAPK signaling pathway is involved in the proliferative effect of danshen and tanshinone IIA on Schwann cells.

## 4. Discussion

The combination of herbal medicine and biomedical material science to promote functional recovery of damaged peripheral neurons has received much attention in recent years. Based on our previous study [13, 14], using a silicone rubber chamber filled with a mixture of herbal product and Schwann cells for bridging a 15-mm gap for rat sciatic nerves is a good model to evaluate the capacity for regenerating neurons. Schwann cells produce and release adhesion molecules and trophic factors which are vital requirements for successful nerve regeneration following injury [15]. Danshen (Salvia miltiorrhiza) is a commonly used Chinese herb in mainland China for the treatment of atherosclerosis-related disorders such as cardiovascular and cerebrovascular diseases. It has been reported to have anticancer effect and to inhibit oxysterol-induced endothelial cell apoptosis. 

**Figure 5:**
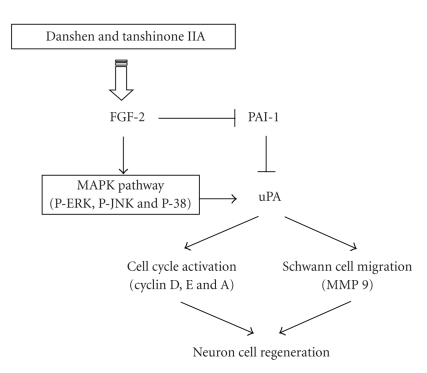
Schematic model of the proliferative effects and nerve regeneration involving MAPK-signaling pathways in Schwann cells and peripheral neurons exposed to danshen and tanshinone IIA. Stimulation of neurons and Schwann cells with danshen and tanshinone IIA activates FGF-2 signaling, leading to the up-regulation of uPA, down-regulating of PAI-1, which in turn activated the cell cycle by increasing cyclin protein (a), (d), and (e), thereby enhancing proliferation and regeneration.

However, whether danshen can enhance the growth of peripheral neurons and Schwann cells is unknown. tanshinone IIA, a monomer extracted from danshen, has been reported to have neuroprotective effects on damage area of the brain. In the present study, treatments of different concentrations of danshen (0, 20, 40, 60, 80, 100 mg/mL) had a proliferative effect on the sciatic nerve regeneration in rats. Consistently, RSC96 Schwann cells treated with concentrations of danshen (0, 20, 40, 60, 80, and 100 *μ*g/mL) and tanshinone IIA (0, 2, 4, 6, 8, and 10 *μ*g/mL) had a dose-dependent proliferative effects. These proliferative effects of danshen and tanshinone IIA were also observed in the results of FGF2-uPA regulating cascade, cell cycle proliferative proteins, and cyclins. In addition, zymography showed that the enzyme activity of extracellular matrix regulating protein (MMP 9) and the migration of Schwann cells were increased after danshen and tanshinone IIA treatment. Furthermore, using chemical inhibitors, the proliferative effects of danshen and tanshinone IIA on RSC cells were found to be P38-, ERK-, JNK-, and MAPK-signaling dependent. These results provide evidence that danshen and tanshinone IIA promote the proliferation of Schwann cells and might enhance the reconstructing of peripheral nerve defects (Figure 5).

 Fibroblast growth factor (FGF), a 16–18 kDa protein and potent trophic factor for a number of tissues, can promotes survival and stimulates proliferation of neural cells and Schwann cells [16–18]. FGF also can promote the extension of early regenerating axons by directly influencing the axons unaccompanied by Schwann cells [19]. This evidence suggests that there is a possible connection between FGF expression and injured-neuron repair. In this study, dissected sciatic nerves and Schwann cells that had been exposed to danshen showed increased levels of FGF-2. Tanshinone IIA also increased the protein expression of FGF-2, indicating that these two compounds have a proliferative effect on neuronal cells. 

Degradation of the extracellular matrix (ECM) is associated with the development of tumor metastasis and tissue growth. One of the key regulators of this process is the serine protease, urokinase plaminogen activator (uPA), which converts the zymogen plasminogen into active plasmin. It is known to act on a wide variety of components of the ECM [20]. Consistent with its role in metastasis, high uPA levels are present in cancers with poor prognosis. The cell proliferation and differentiation process is also an event involving uPA catalytic ECM degradation that increases cell migration by activating MMP 9 [21]. FGF is one of the mediators of uPA activity [22–24]. In contrast, PAI-1 is thought to be the major inhibitor of uPA [20]. Our results show that danshen and tanshinone IIA increased the level of uPA and decreased the level of PAI-1 in a dose-dependent manner.

Mitogen-activated protein kinases (MAPKs) mediate critical signaling pathways that involved in cell proliferation and differentiation [25]. There are three subfamilies of MAPKs: extracellular signal-regulated kinases (ERK), NH_2_-terminal kinases (JNK), and P38 kinase. They are important players in regulating a variety of signaling pathways during organism development, cell cycle regulation, cell proliferation and differentiation, and other cellular and physiological processes [21, 26]. In our study, the results from western blotting analysis and MTT-with inhibitors assay (Figure 1(b) and Figures 4(a)–4(c)) revealed that JNK, ERK and P38 are involved in the proliferative effect of danshen on neuron regeneration both* in vivo* and* in vitro*. Tanshinone IIA had a similar effect on Rsc 96 cells. It is also reported that the highly expressed uPA in the epidermal of damaged tissue is regulated by FGF-2 which affects MAPK kinase kinase (MEKK-1) and MEKK-1's downstream NH_2_-terminal kinases (JNK), and extracellular signal-regulated (ERK) for controlling uPA activity [22–24]. Therefore, danshen and tanshinone IIA-enhanced uPA activity using FGF2 might mediate through the JNK and ERK signaling pathways. 

Due to the requirement in appropriate basal lamina scaffolds for regenerating axons to attach and a sufficient nutritional supply, the Schwann cell plays an important role for axon regeneration. The results of Fujimoto's study [19] demonstrated that the FGF receptor was expressed on the plasma membrane of regenerating axons and that exogenously applied FGF bound to the basal lamina, indicating that the binding of FGF and to its receptor facilitates axon regeneration. During regeneration, Schwann cells supplied the sources of exogenous FGF and the basal lamina to promote nerve regeneration. In our results, danshen and tanshinone IIA-treated Schwann cells demonstrated dose-dependently increased growth rate (Figures 2(a)–2(c)), protein levels of FGF-2, its downstream JNK, ERK and even P38 signaling, and uPA activity with inhibited PAI-1 and the activation of cyclin D, E and B (Figures 3(a)-3(b) and Figures 4(a)–4(c)) at lower doses of two components, indicating that danshen and tanshinone IIA sufficiently enhance the potential of Schwann cell proliferation for neuron regeneration. 

Danshen is a commonly used Chinese medicine. In addition to the discovered health-promoting effects mentioned above, the findings of this study provide another novel danshen neuron regeneration function. However, high doses of danshen and tanshinone IIA suppressed nerve regeneration in Rsc96 Schwann cells, indicating that excessively high concentrations of danshen and tanshinone IIA in the tube or medium may have an adverse effect. Therefore, an appropriate dosage of danshen and tanshinone IIA should be carefully selected for enhancing neuron regeneration. 

Moreover, further studies are needed to investigate whether the combination of Schwann cells or other biomaterials with danshen and tanshinone IIA at appropriate dosage can act additively on the regenerating neuron in the chamber transected into the dissected sciatic nerve in rats.
